# Supplementary material for: Barriers and Facilitators to Implementing Reduced-Sodium Salts as a Population-Level Intervention: A Qualitative Study
Source: Nutrients. 2021 Sep 17;13(9):3225. doi: 10.3390/nu13093225 (PMC8471368; doi:10.3390/nu13093225)
Supplement: Supplementary file 1 [file nutrients-13-03225-s001.zip › nutrients-1347761-supplementary.pdf]

Supplementary File S1. Interview guides

Academic Investigators	Policymakers	Salt manufacturing
	**Overview**	
Can you tell me about your project and describe the reduced-sodium salt intervention in your study?	Were you or your work institute involved in salt reduction strategy in any way? Please tell us about your roles and responsibilities?	Can you tell me about your company (scale, sale area) and the reduced-sodium salt products (formula, price, sales volume) of your study?
Is it a currently ongoing study? When and how long did it last?		Please tell us about your role and responsibility in your company?
What is your responsibility in the study?		Why do you want to produce reduced-sodium salts?
Can you talk about the reduced-sodium salt you used in your study?		Has your company involved in any research or public health activities? If yes, please tell me what the activities are?
Why do you want to implement the reduced-sodium salt intervention?		
	**Reach**	
To what extent do you think reduced-sodium salts are likely to reach the target population for sodium reduction?	Do you know if there is reduced-sodium salt (low-sodium salt) in your country? Do you think what groups are reached?	What the target consumers of your reduced-sodium salt products?
How will the access to reduced-sodium salt be supported?		What groups do you think are not reached and why?
	**Effectiveness**	
What do you perceive as the key benefits of reduced-sodium salt?	Do you think reduced-sodium salt can be an effective approach to reduce population sodium intake? Why or why not?	What do you think is the health impact of reduced-sodium salt?
Do you think reduced-sodium salt can be an	*Prompt*: What settings or types of countries do you	Do you think reduced-sodium salt can be an

effective approach to reduce population sodium intake? Why or why not?<br>*Prompt*: What settings or types of countries do you think reduced-sodium salts will be effective in lowering salt intake?<br>*Prompt*: Are there other strategies that you think would be more appropriate? If so, what?	think reduced-sodium salts will be effective in lowering salt intake?<br>*Prompt*: Are there other strategies that you think would be more appropriate? If so, what?	effective approach to reduce population sodium intake? Why or why not?<br>*Prompt*: What settings or types of countries do you think reduced-sodium salts will be effective in lowering salt intake?<br>*Prompt*: Are there other strategies that you think would be more appropriate? If so, what?
	**Adoption**	
What do you think are the main barriers to consumers' adoption of reduced-sodium salts?<br>Prompt: What suggestions do you have on how to overcome those barriers?<br>How would you improve consumers' adoption of reduced-sodium salts?<br>Has the adoption of organization improved since the initial implementation phase? If so, can you provide some reasons why?	Would you like to use reduced-sodium salts as an intervention? Why or why not?<br>What organizational issues impacted your capacity to carry out these responsibilities in reducing excessive salt intake in your country?<br>What do you think is the overall population's attitudes towards reduced-sodium salt?<br>Can you suggest any strategies that would have increased the participant's	What are the barriers for food industry to adopt the use of reduced-sodium salts? And facilitators?<br>Compare with regular salt/ normal salt products, what are the barriers for consumers to adopt the use of reduced-sodium salts? And facilitators?

	use of the reduced-sodium salts instead of normal salt?	
	**Implementation**	
How would you implement a reduced-sodium salt intervention as a population health intervention?	How would you implement a reduced-sodium salt intervention as a population health intervention? (policy, health education campaign, industry engagement) What do you think is the role of reduced-sodium salts in the overall salt reduction strategy?	Would you like to advocate for reduced-sodium salts? How would you implement a reduced-sodium salt intervention as a population health intervention?
Can you describe any of the critical aspects that facilitated the implementation of the reduced-sodium salt intervention?	Can you describe any of the key aspects that facilitated the implementation of the reduced-sodium salt intervention?	Can you describe any of the key aspects that facilitated the use of reduced-sodium salt?
Can you describe the barriers to implementation of the reduced-sodium salt intervention?	Can you describe the barriers to implementation of the reduced-sodium salt intervention?	What the barriers to increase the use of the reduced-sodium salts instead of normal salt among consumer? And facilitators? What the barriers to increase the use of the reduced-sodium salts instead of normal salt among food industry? And facilitators?
	**Maintenance/Sustainability**	
Do you know whether the participants/organizations are still using or promoting reduced-sodium salt after your study?	Can you describe any experiences you have had trying to scale up reduced-sodium salts? Prompt: How are these interventions maintained? What are the reasons for them continuing or /not continuing??	From your company's perspective, do you want to invest in reduced-sodium salt or use reduced-sodium salt over normal salt in the food industry in future? Why and why not?
Can you suggest any strategies to ensuring the	What is needed to maintain a reduced-sodium salt	

sustainability of the use of reduced-sodium salts over normal salts?	intervention in the long-term?	
	**Other opportunities**	
What do you think the feasible formulations for reduced-sodium salts are?		What do you think are the feasible formulations for reduced-sodium salts?
From your perspective, are there safety issues in the use of reduced-sodium salts?		From your perspective, are there safety issues in the use of reduced-sodium salts?
What opportunities do you have to promote using salt alternatives or substitutes?		Any opportunities to promote using salt alternatives or substitutes?
What are the future research priorities on reduced-sodium salts?		What is the future development plan or business strategy on reduced-sodium salts?
Is there anything we haven't mentioned that you would like to talk about related to the reduced-sodium salt		Is there anything we haven't mention but you want to talk about reduced-sodium salt
